# A qualitative study investigating the barriers to returning to work for breastfeeding mothers in Ireland

**DOI:** 10.1186/s13006-016-0075-8

**Published:** 2016-06-10

**Authors:** Deirdre Desmond, Sarah Meaney

**Affiliations:** Department of Epidemiology and Public Health, University College Cork, Cork, Ireland; National Perinatal Epidemiology Centre, University College Cork, Cork, Ireland

**Keywords:** Breastfeeding, Returning to work, Supports, Qualitative

## Abstract

**Background:**

The World Health Organization (WHO) recommends that mothers exclusively breastfeed for the first 6 months of an infant’s life. In Ireland, currently paid maternity leave is 26 weeks and the expectant mother is required by law to finish work 2 weeks before her expected delivery date. Mothers wishing to exclusively breastfeed for 6 months or longer find themselves having to take holiday leave or unpaid leave from work in order to meet the WHO’s guidelines. The aim of this study is to explore women’s experiences of breastfeeding after their return to work in Ireland.

**Methods:**

This study was carried out utilizing a qualitative design. Initially 25 women who returned to the workforce while continuing to breastfeed were contacted, 16 women returned consent forms and were subsequently contacted to take part in an interview. Interviews were recorded and transcribed verbatim and thematic analysis was employed to establish recurring patterns and themes throughout the interviews.

**Results:**

Women noted that cultural attitudes in Ireland coupled with inadequate or inconsistent advice from health professionals posed the biggest challenge they had to overcome in order to achieve to 6 months exclusive breastfeeding. The findings of this study illustrate that mothers with the desire to continue to breastfeed after their return to work did so with some difficulty. Many did not disclose to their employers that they were breastfeeding and did not make enquiries about being facilitated to continue to breastfeed after their return to the workplace. The perceived lack of support from their employers as well as embarrassment about their breastfeeding status meant many women concealed that they were breastfeeding after their return to the workplace.

**Conclusion:**

While it has been suggested that WHO guidelines for exclusive breastfeeding for 6 months may be unattainable for many women due to work commitments, a different problem exists in Ireland. Mothers struggle to overcome cultural and societal obstacles coupled with inadequate support from health professionals. Encouraging and facilitating women to continue to breastfeed after they return to work will help to normalise breastfeeding within Irish culture and promote continued breastfeeding as a viable option for working mothers.

**Electronic supplementary material:**

The online version of this article (doi:10.1186/s13006-016-0075-8) contains supplementary material, which is available to authorized users.

## Background

Breastfeeding has long been established as the optimal source of nourishment for babies, it is more than just nutrition as it is a living fluid that provides a complex mix of hormones, antibodies and enzymes that are unique to mother and baby which cannot be replicated in formula milk preparations [1]. Since it has been acknowledged that breastfeeding is the superior feeding method for babies, it needs to be protected, supported and promoted even after the mother returns to work [2]. Galtry examined the the impact on breastfeeding of labour market policy and practice in Ireland, Sweden, and the USA, however the number of women who return to work and continue to breastfeed in Ireland is unknown [3]. Lactation breaks are protected in law solely for the period of 26 weeks post birth of the baby [4]. In recent years the statutory maternity leave in Ireland has increased from 18 weeks to 26 weeks, although there was provision for lactation breaks in the workplace of up to 1 h per day until 26 weeks post partum without the loss of pay, it was not revised in the 2004 legislation to reflect the increase of statutory maternity leave, hence the legislative protection for lactation breaks in the work place is not reflective of the current paid maternity leave period [4, 5]. This is the first study into the experiences of mothers who continue to breastfeed after their return to work in Ireland.

The World Health Organization (WHO) recommends that infants should be exclusively breastfed for the first 6 months of life and some breastfeeding should continue up until the age of 2 years old and beyond [6]. While it has been suggested that the WHO guidelines for exclusive breastfeeding for 6 months may be unattainable for many women due to work commitments or personal circumstances, a different problem exists in Ireland, with Irish mothers struggling to even initiate breastfeeding [7]. The rates of mothers who exclusive feeding at 6 months in the Republic of Ireland is 2.4 % [8] and extended breastfeeding is quite uncommon. This is a cultural as well as a societal problem as we do not traditionally nurse infants for this length of time. The recent arrival of an immigrant population has seen the rates of breastfeeding increase in the Republic of Ireland, however research has shown the longer a mother is resident in the Republic of Ireland the less likely she is to breastfeed her infant regardless of her nationality [7].

Ireland had target goals for increased breastfeeding initiation and duration rates by the year 2000, it was envisioned that at least 30 % of all women would be breastfeeding their babies at 4 months at the beginning of the new millennium, a target which it failed to meet [9].

In an Irish context Tarrant and Kearney found that mothers who chose not to breastfeed placed little value on the benefits of breastfeeding, this may be reflecting the overall negative cultural perception of the practice [3], additionally the principal reasons for early cessation appear to be associated with maternal related factors such as having an inadequate breast milk supply to nourish the infant as well as maternal stress and fatigue [3].

The aim of this study is to investigate the barriers to returning to work for breastfeeding mothers and to look at the experiences of mothers who continued to provide breastmilk for their babies after their return to the paid workforce.

## Methods

### Recruitment

Women were contacted initially through a breastfeeding support website (friendsofirishbreastfeeding.ie) and a private lactation consultant. Women who expressed an interest in participating gave permission for their contact details to be provided to the researcher, who were then contacted via email. Of the 25 women initially contacted 12 replied. All women who had continued to breastfeed after their return to work in Ireland were included in the study. All participants apart from one women were Irish by birth. The professions of the women was not asked to protect anonymity. Study information and consent form were forwarded to the participants with a stamped self-addressed envelope so the participants could return the completed consent form. Once consent was received interviews were organised.

Five additional women were recruited through snowballing. This involved women who participated in the study telling other women they knew who had also returned to work while breastfeeding and those women then volunteering to participate in the study. These women contacted the researcher directly and the researcher then followed the protocol as set out above to include these women in the study.

One participant subsequently contacted the researcher and asked that her contribution not be used in the study as she was currently on work experience and was concerned that she may prejudice future career prospects because of her breastfeeding while on work experience. This interview was deleted immediately upon request and no data provided has been included in this study. This brought the final number of participants in this study to 16. The Topic guide was used as an aid during interviews (Additional file 1). To ensure rigour, the transcripts of all recordings and were firstly transcribed, checked by DD, and then reviewed by SM.

A qualitative research methodology was used to conduct this study design. Holloway and Wheeler 2004 argue that peoples perspective and ones interpretation of one’s experiences is more evident through qualitative research [10]. Pope and Mays 2009 make the point that when you go below the surface of interviews and actually analyse what people have said to you things can appear very differently, they also state that the real strength of qualitative research is induction or interpreting the data, because that is where you find the unexpected [11]. Braun and Clarke 2006 argue that qualitative approaches to research are incredibly diverse and complex and that thematic analysis has the benefit of being flexible [12]. Through its theoretical freedom, that is, not being as constrained into a specific framework with which it has to abide, thematic analysis provides a flexible research tool which can potentially provide a rich and detailed, yet complex account of data [12].

### Sample

Data collection commenced at the beginning of April 2014, and ran until the middle of June 2014. Sixteen semi-structured interviews were carried out at times most convenient to the participants. Fifteen interviews were carried out over the telephone and one interview was conducted face-to- face. Sample characteristics are outlined in Table 1. Interviews conducted were between 15 and 56 min in duration and were recorded using a Dictaphone. Each interview was transcribed verbatim by the researcher (DD).

**Table 1:** Profiles of study participants

Participant	Age (years)	Tertiary level education	Number of children	Age of child when returning to work while continuing to breastfeed (months)
A	38	Yes	2	8
B	34	Yes	1	8
C	36	Yes	1	7
D	33	Yes	2	9
E	34	No	1	6
F	24	No	2	8
G	33	Yes	2	7
H	36	Yes	1	8
I	34	Yes	1	10
J	35	Yes	1	10
K	34	Yes	2	6
L	40	Yes	3	11
M	33	Yes	2	6
N	36	Yes	2	11
O	36	No	2	8
P	33	Yes	2	9

### Analysis

Thematic analysis as described by Braun and Clarke [12] was used as the method for data analysis. This involved reading and rereading of the transcribed text to familiarise the researcher with the data and to identify preliminary patterns and themes. This initial step allowed the researcher to allocate basic codes to the themes that were relevant to the research question. The codes were then organised into a hierarchical structure for ease of interpretation and divided into themes and sub-themes by devising a mind map. The mind map was then continually refined until a clear picture of the prominent themes emerged with little overlap between the themes. The themes were then grouped into linked dimensions and perspectives for expansion to provide in depth knowledge of the researchers area of interest. The accounts women gave of their experiences were extremely personal to them yet they were repeatedly echoed in the experiences of other women. This repetition of common experiences although unique but similar eventually gave rise to data saturation [12].

## Results

Four prominent themes emerged from the data, these were Culture, Supports and information provision, Returning to work and Feeding in the workplace.

### Culture

Cultural attitudes towards breastfeeding were a prevalent theme throughout all of the discussions with the women who participated in this study. The range of experiences shared by women included negative comments from family and friends to the pressure of breastfeeding in public. Women described how they experienced negative social perceptions of breastfeeding and perceived acceptable length of time for breastfeeding. This was particularly evident when it came to returning to the workplace after maternity leave. Many women did not disclose they were still breastfeeding to managers or colleagues for fear they would be subject to judgement or criticism for continuing to breastfeed after their return to work.

P: Definitely the feeding in public, so, when, when I was breastfeeding (Baby), I bought a poncho, that was specifically designed to put over the baby while you’re feeding because I was genuinely really uncomfortable feeding in public. I felt like men would look away and they would be really embarrassed and I, kind of, got sick of having to leave the room every time we had family and friends around because, you know, I’d be missing out on all the banter while I’m upstairs feeding (Age 33, breastfed for 12 months).

When asked if her colleagues knew she was breastfeeding participant N said:

N: I don’t know. I wasn’t in that situation much really. I, yeah, I think I probably was a couple of times. I said, for break, I’d say, ‘Would you mind if I go first?’ and like, I suppose, it’s not, I didn’t really feel like it was much people’s business. I started in a new area as well. All to reduce hours. So, really, I didn’t know them as well and I had to, you know, it’s not that I’m a very private person but I suppose it’s my own personal thing and I think feeding them to that big is your own personal thing as well. So, I suppose, I just didn’t, kind of, want everyone, I think I might’ve said it to one or two (Age 36, breastfed for 17 months).

Some women felt that breastfeeding was misrepresented and that Irish culture was not accepting of breastfeeding due to misconceptions and misinformation. Many women felt that normalising breastfeeding would go a long way towards making society more open to the practice.

H: “This country really needs to change its attitude towards breastfeeding, it really does, you know the health factors and everything, and I don’t know why it’s hidden, you have to feel embarrassed and hide it” (Age 36, currently breastfeeding).

When asked about her breastfeeding experience participant L said:

L: Our attitude to breastfeeding is very poor sometimes and I think that women who do breastfeed and continue to breastfeed beyond six months, kind of, feel a bit alienated. I certainly did…I just, kind of, felt that I was holding a bigger baby now and people felt that, people around me felt that, you know, now that I was, the baby was on solids, would I not switch to formula and, you know, I’ve done my job, what would I want continue to breastfeed for? I just found a negative attitude when I was feeding a bigger baby than a smaller baby…we are just so slow in our attitude changing with breastfeeding. It’s painfully slow.

Ridiculously slow, I don’t see any huge difference between when I started 8 years ago and now (Age 40, breastfed for 22 months).

### Supports and information provision

Having a good support network was very important for all the participants, from partners and family to health professionals. All women said that the support they received helped them enormously with both the initiation and duration of their breastfeeding experience. It was also apparent that women who chose to breastfeed were very passionate about it and all of the women interviewed described themselves as “stubborn” or “determined”. They said this helped them to overcome any obstacles they faced.

J: I’ve always done my own thing anyway, I like to think that I’m not easily influenced by people or I am not like that, but it has you know, there is only so much that you can listen to of “oh he’s still hungry, how can he still be hungry?, are you ever going to put him down? He needs to sleep in his cot” there is only so much of that crap you can listen to before you get really angry and it bothers you (Age 35, currently breastfeeding).

The hospital stay was, for most participants of this study, the most difficult time in their breastfeeding experience. Many women felt that the midwives did not give them the attention they needed to feel confident in feeding their baby. All women who had this experience said that it only took one supportive healthcare worker to sit with them and take the time to ensure the baby was latched on properly and the mother was positioned correctly to alleviate their concerns and instil a sense of confidence in the mother regarding her ability to breastfeed. The lack of support and knowledge from health care professionals left a very negative impression on them.

F: I have had friends whose public health nurse was a lactation consultant and she turned around to them and said your baby is not putting on weight you need to go to the shop and get formula, that’s an even bigger let down I think. And she’s a public health nurse who charges (Euro) € 75 an hour for a consult like, but as a public health nurse like she is pushing formula (Age 24, currently breastfeeding).

L: I know, like, plenty of other instances where the, where they could’ve been turned off breastfeeding by just going to the GP and your GP is supposed to be your, your first port of call in the community (Age 40, breastfed for 22 months).

F: Being a new mother I didn’t know what to do, and I was like “when do I feed him? What do I do?” and the midwife said “oh yeah, one second” and she went out of the room and she came back with like 4 different bottles of formula and she was like” Which one do you want?” and I was like, “no no, I want to feed him myself” and she literally stuck his head on my boob and then just walked away (Age 24, currently breastfeeding).

Some women have had relationships breakdown over the lack of support from family members, particularly mother’s in law. The perceived negative response to breastfeeding deeply offended some women and as a result difficulties arose some of which have been thus far irreconcilable.

C: The only family I really had any contact with was my mum and she was really supportive of breastfeeding but I would definitely have got comments from (Partners’) mum, and that have been quite detrimental to our relationship, we don’t talk now (Age 36, currently breastfeeding).

J: I can’t say it didn’t have an effect on me, it made me very sad that my relationship with my parents in law has suffered greatly because I breastfed (Age 35, currently breastfeeding).

The need for practical and emotional support was described by all women to varying degrees however all women indicated that some kind of support was absolutely necessary for breastfeeding mothers. Many women found breastfeeding support groups particularly helpful for practical and professional information. Internet breastfeeding forums were described as a great source for informal advice and sharing experiences with other breastfeeding mothers. A lot of women found peer support very helpful, especially when family support was lacking. Partners and husbands were repeatedly reported as being the greatest support for the mother and all the women who participated in the study spoke of the practical and emotional support the baby’s father provided during the maternity leave period and after the mother’s return to work.

H: I do think you need your husbands or partners support, if you don’t have their support on it, you won’t stick with it, because there are such tough times with the growth spurts and they happen so quickly and they go on for so long, I mean at 8 days I thought I’d never get out of the chair, and it was great that he was there, he could bring me water and sandwiches and food and take her to change a nappy and then bring her back again. Nothing can prepare you for that, no matter how much research you do, no doubt, you do need partner support (Age 36, currently breastfeeding).

M. I think, if people had support, more support from the professionals and also from within their families, it would, it would probably make a lot of difference to people even, even starting, never mind carrying on…I think that people are very, very social creatures and I think they need support from other people. They need to be encouraged all the time that what they’re doing is a good thing, that it’s not weird (Age 33, currently breastfeeding).

### Returning to work

The infants feeding routine was of importance at the time of the mothers return to work. Women spoke of the sense of urgency to get a feeding routine established. Some women described the pressure to get their babies onto a bottle before their return to work resulting in emotional stress and anxiety. While many women in this study returned to work on a full time basis and continued to breastfeed, most of the women took unpaid leave (if they could afford to) and returned later than the statutory 26 weeks maternity leave so their babies had been introduced to solid foods. Women referred to their situation as being “very lucky” or “very fortunate” that they could continue to breastfeed after their return to work due to their infant needing less feeds during the day and so they were able to provide some breast milk through expressing which could be given to the infant in a cup or a bottle and the pressure of on demand feeding had ceased. While this may have been fortuitous for the women concerned it was as a result of returning to work when the infant was older than the 6 months that the WHO recommends exclusive breastfeeding for and their infant had begun on solid foods which naturally decreased the need for breast milk, rather than employers putting provisions in place to support breastfeeding employees.

E: It annoys me, it annoyed me that I felt under pressure to get her on bottles, I felt really pressurised a month before I was going back, I remember I felt under pressure the month before, because she still was very finicky with the bottles, very finicky, I felt pressure yeah, I felt it, I knew it, I knew it in my own heart and soul she was in trouble if she would not take the bottles, trying to explain that to my employer, saying, I can’t come back to work because I’m breastfeeding, I feel, I’m an alien to him basically, I just, like he’s a man in his, what? Late 50s maybe, I just don’t think he’d understand (Age 34, currently breastfeeding).

M: So, that was the thing that really worried me about going back was, like, well, I don’t want to be just starting her on solids and throwing her into formula and all of that and, you know, kind of, worry about her nutrition and all that kind of thing but at the same time try to manage a job and actually a new job, I’ve started a new work. So, there was a lot of things to try and juggle…I was very upset by it and he was, he was 7 months at that stage because I actually weaned him way before because everyone kept saying to me, ‘Oh, you don’t know if he’ll take the bottle’, and, you know, ‘You really have to push it onto him’, you know, all this kind of stuff…So, I forced him into it before he was probably ready and, yeah, it was very upsetting for me. I wasn’t ready. I didn’t think he was ready either but I, I did it anyway. So, I would have much preferred to have carried on especially since I was only working 2 days a week…It was just a lack of information on my point or even just a lack of understanding that I could carry it on (Age 33, currently breastfeeding).

When women went back to work they found it was necessary to alter their feeding routines in order to fit in with their work schedule. While this was to be expected, some women found the transition from breastfeeding on demand to trying to get the baby onto a bottle for feeding during the day very difficult.

E: I got to the 6 months and at the 6 months she had to have the bottles during the day because I had to be at work, I asked work before I went back actually could I start at 10, I used to have to be in for 9.15 so I asked them could I come in for 10 because she was still feeding in the morning so I had to feed her in the morning before she had the bottle at 10 or 11 o’clock. So I asked them, and they were fine with that, there was no problem, I literally cut back my hours to do it (Age 34, currently breastfeeding).

G: Yeah, I was demented now from it to be honest, it was a huge relief in one way when he decided to take a bottle, really felt a pressure being lifted off me, then there was another side of it where I felt a bit bad for him that he was missing out, like there was another few weeks of goodness left in me that he lost out on, but I suppose I had no choice (Age 33, breastfed for 10 months).

The emotional pressures and anxieties the woman experienced through this transition period became very clear. The expression of breast milk was an issue for many women, it was reported that many women disliked pumping breast milk describing the process was either unpleasant or undignified or both. The women in this study all described difficulties with lack of facilities to express and store breast milk while at work. The storage of the expressed milk was also an issue for some women who had no facility to store the milk in the workplace and had to bring ice packs and cooler bags to work with them in order to store their milk. They also spoke of the lack of time available to them to express milk during their working day. Some thought of it as very time consuming and said the amount of preparation including the sterilisation of equipment and surface areas made the process a chore.

G: Well I told my manager, I rang her a week or two before I went back to work, they knew I had taken the 6 weeks unpaid, tried to get the child onto a bottle but I rang her a week or two before I was due to go back and I told her I couldn’t get him onto a bottle and I would still be breastfeeding when I went back to work and I would have to pump during the day. They said that was fine, no problem but there is no such thing as anyone coming to you and saying “look you go away now and take your break to pump”, it wasn’t like that, it was like well you can fit it into your own day. Oh, Oh my God, it was terrible, it was so upsetting, you know when, you know the only way I could describe it was that I was totally and utterly consumed with this issue, oh Jesus, I was actually losing my mind (Age 33, breastfed for 10 months).

P: I think it’s definitely, it has to be a personal choice. I’m not sure there’s anything you can do to, you know, like, several of my friends would’ve said they would like to continue but it’s not possible at their work. So, one of my friends is a teacher. And there was just nowhere in her school that she could have some privacy to pump (Age 33, breastfed for 12 months).

### Feeding in the workplace

Some women who took part in this study experienced negative attention or a lack of support due to their continued breastfeeding while being back at work. One woman in this study actively felt hostility towards her from her colleagues when she went to express milk. She reported colleagues ringing her on her mobile phone to see where she was when she went to express milk and also feeling that people were talking about her behind her back complaining that she left her duties while she went to express milk.

The financial implications of taking extra unpaid maternity leave was an issue for some women and they had to return to work at the end of the statutory maternity leave, these women found their return to the workplace particularly difficult.

E: I couldn’t like, it literally would not have been worth my while to go back on, I mean losing the hours until 10 o’clock killed me as well, every hour counts at the end of the day you know, it did you know like, but I didn’t have a choice in doing it, yeah, because I couldn’t afford to take the 16 weeks [unpaid leave], there was no way (Age 34, currently breastfeeding).

B: I suppose it is a lot of stress then going ‘God I have to go and pump now’ so in terms of legislation I think two things, I think ideally maternity leave being longer. The reason why I’m going back earlier this time is, because the state pays so little. But this time round, financially, I’m gonna find it hard to even take the 6 months to be honest so my plan is to do a day or two a week, maybe when the baby is around 4 months (Age 34, breastfed for 20 months).

When discussing feeding in the workplace women described the lack of facilities and supports as a major barrier to continued breastfeeding. For the mothers who did need to express milk during their working day all experienced difficulties with a space to pump milk and finding the time to express.

I: better facilities and maybe a room, going to the toilets is not on, it’s not hygienic and it’s embarrassing (Age 34, breastfed for 2 years).

F: No, there is the canteen and the office in work but I’ve been told I can use them but there is not a specific breastfeeding room with a lock on the door like. I have to put a sign up, there is no actual lock on the door so I put a sign up and put a heavy chair behind the door so no one can actually open it (Age 24, currently breastfeeding).

The attitudes of colleagues and the stigma surrounding extended breastfeeding was an issue for some women. Mothers felt they could not be forthcoming about their decision to continue to breastfeed after their return to work with their colleagues as they felt they would have been the subject of workplace gossip and negative attention.

G: If there was something that was there in black and white and you could say look, listen lads, it’s down her in black and white and I am entitled to go for 20 min, two or three times a day or whatever it is there is definitely more of a backup for you that you can say I’m entitled and you can forget their tut tut tut “where are you going?”. So like I used to pump at work but where I would have liked to pump twice a day at work I only ever got to pump once a day and at that it was a case of “oh, where she’s off to know with her pink bag?” it was, it was comments like and I am sure they were talking about me behind my back (Age 33, breastfed for 10 months).

When speaking about her manager’s way of dealing with her expressing milk at work participant O said:

O: Now, he didn’t speak to me himself. He got, like, one of his team members to speak to me about it, just organising everything and she asked about where would I store the milk and I said, ‘Well, I’m just going to put it in the fridge. So, you know, don’t worry about it.’ Because I thought she was asking out of concern for me, and where I was going to store it and she said that himself had basically said, ‘Oh, he’s just concerned that some people might have, you know, a bit of an issue with breastmilk being stored in the fridge.’… So, a lot of people that are pre-empting their employers, ‘I won’t ask because they’re just going to say, ‘no’, and then it might be embarrassing or it might be annoying’, whereas if they were to just say, you know, there’s no legal requirement after the 6 months (Age 36, currently breastfeeding).

All women said that having legislative protection for lactating mothers that continued after the 26 weeks post-delivery that is currently in place would help women to feel more confident regarding approaching employers about providing adequate facilities and breaks to support women to continue to feed after their return to work.

C: I mean my son was 2 weeks overdue, and obviously I was off for the 2 weeks before he was due so I was off for nearly a month before he came, and then really I would have had to go back when my 26 weeks was up and he would have just been gone 5 months old. It’s not long enough, really not long enough. And paternity leave is non-existent, so what do you do? (Age 36, currently breastfeeding).

I: Legal protection for women to feed after their return to work, I work in a private company and they have no obligation to allow me to take breaks to pump or feed (Baby) if it was a legal right I would be much more confident in saying it to my boss, I am taking the break I am entitled to feed my child, if it’s the law you have full protection, if it is just up to the company you are at their mercy, and they have all the control, but if it’s your legal right you have protection. Just one more thing, the maternity leave is not long enough here, the baby is still feeding constantly when you have to go back to work so it is not possible to feed for 6 months (Age 34, breastfed for 2 years).

## Discussion

A review of the literature was undertaken to determine the rates and duration breastfeeding in the Republic of Ireland as well as the rates of women who return to work while breastfeeding in the Republic of Ireland. While there are numerous articles relating to breastfeeding and its benefits, the literature in relation to breastfeeding for 6 months or more in an Irish context is very scant. “Although low breast-feeding rates, both traditionally and currently, remain a national public health issue, it is a further concern that very limited high-quality breast-feeding and infant feeding data exist in Ireland” [3]. There was no literature in relation to the rates of women who return to work while continuing to breastfeed in the Republic of Ireland.

The results of this study show that women in this study in Ireland who choose to breastfeed are often met with an array of challenges. Social stigma, lack of consistent or conflicting advice from healthcare practitioners and inadequate maternity leave appeared to make breastfeeding an unattractive feeding choice for many mothers. Women who chose to breastfeed for 6 months or longer all demonstrated a fierce determination to do so, overcoming all of the challenges they encountered.

The practice of breastfeeding has been lost to generations of Irish women due the steep decline in breastfeeding rates from 64 % in the 1950s to 16 % in 1975 as formula feeding has become an accepted feature of life in Ireland [3]. The Republic of Ireland currently has one of the lowest breastfeeding rates in Europe with only 55.4 % of all women initiating breastfeeding, 42 % breastfeeding at 48 h or at discharge and 19 % continuing to breastfeed at 2–3 months [3]. Our study indicated that information provision from health professionals varied greatly and many women were given inconsistent or conflicting advice. Mothers found that some midwives were encouraging and supportive of breastfeeding, others recommended formula feeding. The advice from public health nurses often conflicted with the advice the mother received in the hospital resulting in mothers seeking the services of private lactation consultants or Le Leche League support groups for advice. A number of women found peer advice very helpful, particularly when professional advice was absent. Our data suggest that health professionals need to give encouraging and consistent advice to expectant mothers’ right through pregnancy and after birth in order to inspire confidence and self-efficacy in women regarding their ability to breastfeed.

Our findings show that there is a lack of social approval for breastfeeding mothers and breastfeeding in the workplace is not encouraged. This finding is in line with Gatrell who stated that mothers faced difficulty as breastfeeding within the workplace is considered ‘taboo’ [13]. She further noted that for mothers to comply with workplace requirements they were either obliged to cease breastfeeding or to conceal breastfeeding activities [13], this was also the case for some women in this study. Lack of supportive work environments, lactation facilities and the period of paid maternity leave have all been cited as barriers to breastfeeding in the workplace [14]. Similarly, the women in this study experienced emotional pressures and anxieties when they were due to return or had returned to the workplace. The financial implications of taking extra maternity leave was an issue for some women and they had to return to work at the end of the statutory maternity leave. Women found the lack of facilities and supports for breastfeeding mothers in their workplace as a major barrier to continued breastfeeding. Hawkins et al., suggests that mothers who are employed on a full time basis are more likely to initiate breastfeeding if their employer offered supportive breastfeeding arrangements [15]. Greater efforts are needed to positively promote breastfeeding at all levels of society and reverse the negative attitudes towards breastfeeding in Ireland [8].

### Strengths and limitations

This study was strengthened by the use of a qualitative design study. The information gathered was very rich and detailed but also very personal in nature. This divulging of sensitive information was facilitated through the conversation between the participant and the researcher and yielded very interesting data relating to the women’s beliefs and opinions as well as their experiences. Although it was a relatively small sample size the experiences of the women shared many similarities. In addition, the constant comparative method was utilised [16] and following these interviews no additional information or new themes were rasised by participants. This in turn lead the researchers to believe that data saturation had been reached [12]. However qualitative data has its limitations as the data are not generalizable [16]. Although the recounting of experiences is subjective, theparticipants from this study shared many unique though similar experiences. The findings of this study are similar to quantitative studies reported elsewhere and would be interesting to see if similar findings would be found if the study were repeated elsewhere. This study was limited by the lack of previous research carried out investigating mothers who continue to breastfeed after their return to work in Ireland. The lack of comparable data into challenges faced by women in Irish workplaces who wish to continue to breastfeed after their return to work suggests that further investigation into this area is needed.

## Conclusion

Upon their return to the workplace many women find that they do not have any legislative protection for lactation breaks to either express breast milk to feed at a later time or to feed their infant on the premises if facilities are available. Lactation breaks are protected in law solely for the period of 26 weeks post birth of the baby [5]. In recent years the statutory maternity leave in Ireland has increased from 18 weeks to 26 weeks, although there was provision for lactation breaks in the workplace of up to 1 h per day until 26 weeks postpartum without the loss of pay, it was not revised in the 2004 legislation to reflect the increase of statutory maternity leave, hence the legislative protection for lactation breaks in the work place is not reflective of the current paid maternity leave period [4, 5]. Extending paid maternity leave and introducing paid lactation breaks for all women who want to continue to breastfeed may encourage more women to feed after their return to work.

## Additional file

Additional file 1: Topic guide. (DOCX 13 kb)
