# Supplementary material for: A qualitative study investigating the barriers to returning to work for breastfeeding mothers in Ireland
Source: Int Breastfeed J. 2016 Jun 10;11:16. doi: 10.1186/s13006-016-0075-8 (PMC4902923; doi:10.1186/s13006-016-0075-8)
Supplement: Additional file 1: — Topic guide. (DOCX 13 kb) [file 13006_2016_75_MOESM1_ESM.docx]

**Topic Guide: Barriers to returning to work for breastfeeding mothers.**

Demographics: Name, age, previous pregnancies and employment status.

Breastfeeding History:

How long did the woman nurse her Infant? Was it exclusive breastfeeding?

What influenced her decision to breastfeed?

Breastfeeding Experience:

How did the woman feel about breastfeeding? What challenges did she experience?

Did she receive appropriate support?

Did other women in her social network breastfeed?

Ceasing Breastfeeding:

What factors influenced her decision to discontinue breastfeeding? Did going back into the workplace play a part in this decision? How did she feel about ceasing breastfeeding?

Returning to the workplace:

How long after giving birth did she return to the workplace?

How many hours per day/week did the woman work upon her return to the workplace?

How did she experience her return to the workplace?

Were breastfeeding supports in place within her workplace?

How did she combine breastfeeding with returning to workplace?

Second and Subsequent pregnancies:

With second and subsequent pregnancies did the knowledge the woman gained from her prior breastfeeding experience influence her decisions regarding continuing to breastfeed for longer, particularly after her return to work?

Thoughts or Opinions:

What supports are needed to encourage women to breastfeed after their return to work?
